# Adaptation and Validation of the 8-Item Depression, Anxiety, and Stress Scale (DASS-8) in Peruvian School Adolescents

**DOI:** 10.3390/bs16020269

**Published:** 2026-02-12

**Authors:** Liset Z. Sairitupa-Sanchez, Sandra B. Morales-García, Oriana Rivera-Lozada, Wilter C. Morales-García

**Affiliations:** 1 Unidad de Psicología, Escuela de Posgrado, Universidad Peruana Unión, Lima 15102, Peru; lisetsairitupa@upeu.edu.pe; 2 Medicina Humana, Universidad Señor de Sipán, Chiclayo 14001, Peruriveraoriana@uss.edu.pe (O.R.-L.); 3 Dirección General de Investigación, Universidad Peruana Unión, Lima 15102, Peru; 4 Facultad de Teología, Universidad Peruana Unión, Lima 15102, Peru

**Keywords:** DASS-8, mental health, validation, adolescents, school students, Peru

## Abstract

Background: Depression, anxiety, and stress are common disorders among school adolescents, affecting their emotional well-being and academic performance. In Peru, there is a lack of brief, validated instruments to detect these symptoms in educational settings, which limits timely intervention. Objective: To adapt and validate the Spanish version of the 8-item Depression, Anxiety, and Stress Scale (DASS-8) in Peruvian school adolescents. Methods: A total of 801 Peruvian adolescents between 11 and 18 years of age (M = 14.04; SD = 1.54) participated. An instrumental design was used, including translation and cultural adaptation of the DASS-8. Confirmatory factor analysis (CFA), reliability estimates (α and ω), and factorial invariance testing by sex, stage of adolescence, type of institution, and place of residence were conducted. Results: The model showed excellent fit (χ^2^ = chi-square)(17) = 48.000, Comparative Fit Index (CFI) = 0.99, Tucker–Lewis Index (TLI) = 0.98, Root Mean Square Error of Approximation (RMSEA) = 0.05, and Standardized Root Mean Square Residual (SRMR) = 0.01. The subscales demonstrated high reliability: Depression (α = 0.90; ω = 0.90), Anxiety (α = 0.90; ω = 0.90), and Stress (α = 0.87; ω = 0.87), with an overall α of 0.95. Strict invariance was supported by gender and type of institution, and scalar invariance was supported across stages of adolescence and place of residence. Conclusions: The Spanish DASS-8 is a valid, reliable, and useful tool for screening emotional symptoms in Peruvian school adolescents. To the best of our knowledge, this is the first study to evaluate the psychometric properties of the DASS-8 in Latin American school adolescents.

## 1. Introduction

Mental health has become an area of growing concern in medicine and public health, both globally and locally (McGorry et al., 2022; Wainberg et al., 2017). Specifically, the mental health of school-aged children and adolescents has emerged as a priority, given the vulnerability of this population to developing psychological disorders that can have lasting effects on their well-being and development (Garcia-Carrion et al., 2019). As psychosocial challenges such as academic pressure, cyberbullying, and other stressors increase, the need for appropriate assessment tools to diagnose and monitor the mental health of school students becomes imperative (Hellfeldt et al., 2020; Phan et al., 2022). Among mental health disorders, depression, anxiety, and stress are among the most prevalent in youth populations (Mohler-kuo et al., 2021; Sandal et al., 2017). Beyond their impact on emotional well-being, these disorders are related to disturbances in other domains of physical and mental health, such as sleep hygiene, where anxiety has been associated with poor sleep patterns and unhealthy sleep behaviors among adolescents and university students (Peng et al., 2025), as well as with difficulties in behavioral regulation, including behavioral addictions such as internet addiction, which can exacerbate emotional symptoms and academic difficulties (Liu et al., 2024).

Depression is conceptualized as a mood disorder characterized by a persistent state of sadness, emptiness, or irritability, accompanied by cognitive and somatic changes that significantly impair academic, social, and family functioning (Chand & Arif, 2023). From a dimensional perspective, it involves dysphoria, hopelessness, devaluation of life, self-deprecation, loss of interest or engagement, anhedonia, and inertia, forming a pattern of low positive affect and motivational withdrawal (Bados et al., 2005; Lovibond & Lovibond, 2005). Anxiety is defined as an emotion characterized by feelings of tension, worried thoughts, and physiological changes (e.g., tachycardia, muscle tension) associated with the anticipation of a future threat, whether real or perceived (American Psychiatric Association, 2012; Juby & Felman, 2023). Anxiety disorders are described as conditions in which fear and worry are excessive and persistent, accompanied by autonomic hyperarousal and avoidance behaviors that lead to functional impairment (American Psychiatric Association, 2012; Parlakyıldız, 2025). In the DASS, anxiety is conceived specifically as a pattern of physiological hyperarousal (autonomic arousal), effects on skeletal musculature, situational anxiety, and anxious affects, emphasizing the somatic fear component rather than diffuse cognitive worry (Henry & Crawford, 2005; Lovibond & Lovibond, 2005). Psychological stress, in turn, is understood as a relational process in which the individual appraises environmental demands as exceeding their coping resources and threatening their well-being (Biggs et al., 2017; Lazarus & Folkman, 1984). In the DASS, stress is conceived as a level of nonspecific chronic arousal that includes difficulty relaxing, nervousness, irritability, a sense of being “tense,” and a tendency to overreact to everyday demands (Edimansyah et al., 2008; Lovibond & Lovibond, 2005). During adolescence, these three domains are associated with suicidal ideation, academic overload, family conflict, sleep problems, and risk behaviors, making their systematic assessment in school adolescents a public health priority (Beirão et al., 2020; Thapar et al., 2012; WHO, 2025).

Globally, mental health disorders among school students are increasing in prevalence (Hassan et al., 2023). According to the World Health Organization, it is estimated that 1 in 7 adolescents between 10 and 19 years of age experience a mental health problem, although, in many cases, these problems are neither recognized nor treated (OMS, 2021). Adolescence is a stage of major physical, emotional, and social changes; factors such as poverty, abuse, violence, and social pressures can make adolescents particularly vulnerable to these problems (OMS, 2021). In the Peruvian context, recent evidence shows a high burden of emotional problems among children and adolescents: the Epidemiological Study of Mental Health in Children and Adolescents in the Context of COVID-19 reported that more than one-third (33.2%) of school students aged 6 to 17 were at risk of presenting a mental health problem, and that 29.6% of adolescents aged 12 to 17 were at risk of having a mental or emotional health problem (MINSA, 2020). Complementarily, a national youth report indicated that nearly one-third of Peruvian youth aged 15 to 29 years reported having experienced a mental health problem in the last 12 months (Ministro de Educación, 2021; SENAJU, 2023). In addition, a considerable proportion of children and adolescents have been found to present mental health-related conditions linked to socioeconomic, cultural, and educational factors specific to the country (MINSA, 2020). For example, academic stress, lack of access to mental health services, and the stigma associated with mental disorders may contribute to the rising prevalence of these problems among Peruvian school students (Valladares-Garrido et al., 2023; C. Zhang et al., 2022). Taken together, depression, anxiety, and stress in school populations impair quality of life, academic performance, and interpersonal relationships, and in extreme cases may be associated with suicidal ideation and attempts (Deng et al., 2022; Ramón-Arbués et al., 2020; Schulte-Körne, 2016; Zheng et al., 2023).

These data underscore the urgency of having adapted and validated tools for the early detection and monitoring of mental health disorders in Peruvian school students. Although several instruments are available, cultural and linguistic adaptation is essential to ensure the accuracy and relevance of these measures in the Peruvian context (Ferrando & Lorenzo-Seva, 2014). The Depression Anxiety Stress Scale (DASS), in its DASS-42 and DASS-21 versions, has been a benchmark for measuring these three emotional constructs in diverse populations. As has been extensively discussed, adaptation of the DASS-21 in different cultures has yielded notable structural variations. In distinct cultural contexts, the process of adapting and validating a scale involves linguistic, semantic, and conceptual considerations. For example, cultural manifestations of depression or anxiety may vary according to the norms and values of a given society. In the case of the DASS-21, studies in Turkey and Brazil have revealed divergent factor structures, reflecting how symptoms of depression, anxiety, and stress are expressed and perceived in these cultures (Silva et al., 2016; Yıldırım et al., 2018).

In this context, short scales have gained prominence because they allow for more agile assessment, reduce the cognitive burden on respondents, and promote higher response rates, especially in clinical and school settings with limited resources. They are also well suited for large-scale population-based research and rapid mental health screening (Ali et al., 2021, 2022c). One of the most widely used tools internationally is the Depression Anxiety Stress Scale (DASS), in its 42-item and 21-item versions (DASS-42, DASS-21), designed to differentially assess symptoms of depression, anxiety, and stress under the tripartite model proposed by Lovibond and Lovibond (2005). However, multiple studies have pointed to problems in the factorial structure and measurement invariance of the DASS-21 across cultural contexts, which has driven the development and evaluation of abbreviated versions such as the DASS-12, DASS-9, and, more recently, the DASS-8 (Kyriazos et al., 2018; Lee et al., 2019; Yusoff, 2013; Z. Zhang et al., 2024). In a sample of medical school applicants, the 12-item and 9-item versions (DASS-12, DASS-9) showed better factorial fit and stronger psychometric properties than the 21-item version, supporting the use of briefer formats for screening psychological distress in highly demanding educational contexts (Yusoff, 2013). Similarly, in a general adult Greek population, the DASS-21 and DASS-9 demonstrated a stable three-factor structure, adequate reliability, and strict measurement invariance by gender, whereas broader multicultural studies have documented that scalar and strict invariance may not be fully sustained across countries, underscoring the need to validate these scales in specific contexts (Kyriazos et al., 2018; Scholten et al., 2017; Zanon et al., 2021). In the Asian context, psychometric evaluation of the DASS-21 and DASS-12 in a Korean population showed adequate construct validity, good convergent and discriminant validity, and high internal consistency, concluding that the DASS-12 may be particularly appropriate in care settings with limited time (Lee et al., 2019). More recently, in a large sample of Chinese students, a three-factor structure and strict invariance by sex and educational level were confirmed. Network analyses further showed that symptoms of depression, anxiety, and stress form highly interrelated symptom clusters, which reinforces the relevance of shorter versions focusing on the most central and representative items (Z. Zhang et al., 2024). Within this framework, the DASS-8 has emerged as an empirically derived ultra-brief version that retains the original tripartite structure and has demonstrated a robust factorial structure, high reliability (α ≈ 0.90–0.94), and adequate psychometric properties in various population groups, such as healthy adults in the United States, Australia, and Ghana (Ali et al., 2022c), family caregivers of people with dementia in Italy and Switzerland (Ali et al., 2022a), and women with chronic pelvic pain in Australia (Ali et al., 2022a). In addition, these studies have shown that the DASS-8 can achieve measurement invariance by gender, age, and other sociodemographic variables, although invariance across countries is not always complete, further highlighting the importance of examining its structure and invariance in new populations, such as Latin American school adolescents (Ali et al., 2022a, 2021, 2022c).

Peru, with its cultural and socioeconomic diversity, presents unique challenges and opportunities for the adaptation of mental health tools (Feitelson et al., 2023). Peruvian school adolescents face additional challenges, such as the transition toward modernity, the preservation of Indigenous traditions, and socioeconomic pressures, which may influence the presentation and prevalence of mental health disorders. Despite growing concern about mental health in this population, gaps remain in the availability of adapted and validated instruments for school adolescents (UNICEF, 2023). In this context, adapting and validating the DASS-8 may provide health and education professionals with a brief and robust tool for the early identification of depressive, anxiety, and stress symptoms in educational settings.

Accordingly, the aim of this study was to translate, culturally adapt, and evaluate the psychometric properties of the Spanish version of the DASS-8 in Peruvian school adolescents.

## 2. Methodology

### 2.1. Design and Participants

A quantitative, cross-sectional, instrumental design was used, which is appropriate for studies of adaptation and validation of psychometric instruments, as it allows examination of the internal structure and metric properties of scales in a defined sample (Muñoz et al., 2019). Non-probability convenience sampling was employed (Otzen & Manterola, 2017). The sample was drawn from two regular basic education institutions in Peru, one publicly managed and one privately managed, both serving primary and secondary school students in urban and urban–rural contexts. Inclusion criteria were: (a) being enrolled in one of the participating institutions in 5th or 6th grade of primary school or in any grade of secondary education; (b) being between 11 and 18 years of age; and (c) having informed consent from a parent or legal guardian and assent from the student. Questionnaires with incomplete information were excluded. The sample size was determined using a specialized online calculator for structural equation modeling (Soper, 2024). This tool takes into account multiple variables, such as the number of observed and latent variables in the model, the expected effect size (λ = 0.30), the desired level of statistical precision (α = 0.05), and the level of statistical power (1 − β = 0.95). Although the tool suggested a minimum sample of 220 school adolescents, the study ultimately included 801 Peruvian school adolescents, aged between 11 and 18 years (M = 14.04; SD = 1.54). Most participants identified as boys (50.6%), were in early adolescence (38.1%), attended public educational institutions (69.7%), lived in urban areas (78.8%), and were enrolled in secondary education (89.9%). With respect to grade level, second year of secondary school predominated (28.2%), followed by first year (25.0%) (Table 1).

### 2.2. Instruments

Depression, Anxiety, and Stress: The 8-item Depression, Anxiety, and Stress Scale (DASS-8) was used. This is an abbreviated, validated version of the DASS-21 (Ali et al., 2021) that assesses depression, anxiety, and stress through 8 Likert-type items (0 = not at all, 3 = very much). Previous studies have shown high internal consistency (α ≈ 0.90). The DASS-8 has been validated in both clinical samples and the general population and has shown a high correlation with the original DASS-21, providing evidence of its predictive and discriminant validity.

The Spanish translation of the DASS-8 followed a carefully designed cultural adaptation process to ensure linguistic and conceptual equivalence between the original and the translated version (Beaton et al., 2000). This process was carried out in four key stages:

1. Two bilingual Spanish translators, both native speakers, independently translated the DASS-8 into Spanish. The two resulting versions were compared, and an initial consensus version was developed, combining the best elements of both translations.
2. The consensus version in Spanish was then back-translated into English by two native English speakers who were also fluent in Spanish but unfamiliar with the DASS-8 scale. The purpose of this stage was to ensure that the Spanish translation maintained the original meaning and intent of the English version.
3. A committee of experts, consisting of two psychologists and one educator, reviewed both the Spanish translation and the back-translated English versions. Based on this review, a preliminary version of the DASS-8 in Spanish was developed.
4. The preliminary version was administered to a focus group of 8 participants to evaluate its comprehension, readability, and cultural appropriateness. No interpretation problems were identified, and no linguistic adjustments were necessary. From this process, the final version of the instrument in Spanish emerged, named the 8-Item Depression, Anxiety, and Stress Scale (DASS-8-S), which is presented in Table 2.

### 2.3. Procedure

The study was conducted in accordance with strict ethical principles and was approved by the Ethics Committee of Universidad Peruana Unión (code 2025-CE-EPG-00094). Prior to data collection, formal permission was obtained from the principals of participating in educational institutions, ensuring that the procedures were appropriate for the school context. Subsequently, written information was sent to parents and legal guardians along with the informed consent form, which detailed the objectives, procedures, minimal risks, and confidentiality safeguards of the study. Only students whose parents or guardians signed the informed consent form were invited to participate. Before administering the questionnaire, the nature and voluntary nature of the study were explained to the adolescents in clear, age-appropriate language, and their informed assent was obtained. It was emphasized that they could leave questions unanswered or withdraw at any time without academic consequences. The questionnaires were administered in person during school hours, in regular classrooms at the two participating educational institutions. Administration was carried out by a team of psychologists and the authors of this study, who had been previously trained in standardized administration of the instrument. They provided uniform instructions, supervised the assessment environment (quiet conditions, sufficient time), and addressed comprehension questions without prompting or suggesting responses. Teachers did not participate in the direct supervision of the test in order to reduce social desirability bias and preserve the perception of confidentiality. During administration, the test administrators visually reviewed each questionnaire to identify unanswered items. In cases of omissions, students were invited to check whether they wished to complete their responses with their decision always being respected. This procedure minimized the presence of missing data. The nonresponse rate for DASS-8 items was negligible; therefore, imputation techniques were not required, and psychometric analyses were conducted with the full valid sample.

### 2.4. Data Analysis

An initial descriptive analysis of the items of the Spanish version was conducted, taking into account the mean, standard deviation, skewness, and kurtosis, as well as the corrected inter-item correlations. Skewness (g^1^) and kurtosis (g^2^) values were evaluated according to the acceptability criterion of being within the ±1.5 range (George & Mallery, 2003). In addition, corrected item–total correlation analysis was used to identify and exclude items with r(it–c) ≤ 0.30, or in cases where multicollinearity was detected (Kline, 2016). No missing values were observed in the DASS-8-S items in the database; thus, all analyses were carried out with the full sample, without the need for imputation procedures.

Subsequently, confirmatory factor analysis (CFA) was conducted to test the factorial structure of the scale, using the robust maximum likelihood (MLR) estimation method, which is appropriate for Likert-type data that may deviate from multivariate normality (Brown, 2015; Finney & Distefano, 2013; Muthen & Muthen, 2017). This estimator corrects the χ^2^ statistic and standard errors under non-normality; therefore, no additional procedures for outlier removal were applied, assuming that extreme scores formed part of the actual continuum of emotional distress severity in a community sample. Model fit indices included the chi-square (χ^2^), the Comparative Fit Index (CFI), and the Tucker–Lewis Index (TLI), with recommended values ≥ 0.95, as well as the Root Mean Square Error of Approximation (RMSEA) and the Standardized Root Mean Square Residual (SRMR), with suggested values ≤ 0.08 (Kline, 2016; Schumacker & Lomax, 2016). Internal consistency of the scale was evaluated using Cronbach’s alpha and McDonald’s omega coefficients, considering values above 0.70 as adequate (McDonald, 1999).

To examine measurement invariance (MI) of the scale by gender, stage of adolescence, type of educational institution, and place of residence, multigroup CFAs were conducted following a hierarchical sequence of models: configural, metric, scalar, and strict. Invariance was established when changes in the CFI (ΔCFI) were less than 0.010 between successive models (Chen, 2007). Additionally, an explanatory model was developed using structural equation modeling, applying the same fit criteria and the MLR estimator.

All statistical analyses were performed in RStudio (RStudio Team, 2018), version 4.1.1 of R (R Foundation for Statistical Computing, Vienna, Austria; http://www.R-project.org, accessed on 1 July 2025). The “lavaan” package (version 0.6.19) was used for confirmatory factor analysis and structural equation modeling (Rosseel, 2012).

## 3. Results

### 3.1. Descriptive Statistics of the Items

The results of the descriptive analysis for the DASS-8 items show means ranging from 0.98 to 1.17. The item with the lowest mean was item 6, with a value of 0.98, while the items with the highest means were items 2 and 4, both with a mean of 1.17. The standard deviations ranged from 1.15 to 1.22, indicating moderate variability in participants’ responses. Regarding skewness (g^1^) and kurtosis (g^2^) indices, all items presented values within the acceptable range of ±1.5. The distributions showed slight positive skewness (values between 0.53 and 0.78) and moderate negative kurtosis (values between −0.98 and −1.30), suggesting that the data have a relatively normal shape and are suitable for parametric analyses. The corrected item-total correlations {r(i-tc)} were high for all items, with values ranging from 0.79 to 0.85, indicating that each item maintains a strong relationship with the total scale and contributes significantly to its internal consistency (Table 2).

### 3.2. Confirmatory Factor Analysis and Reliability

**Figure 1:**
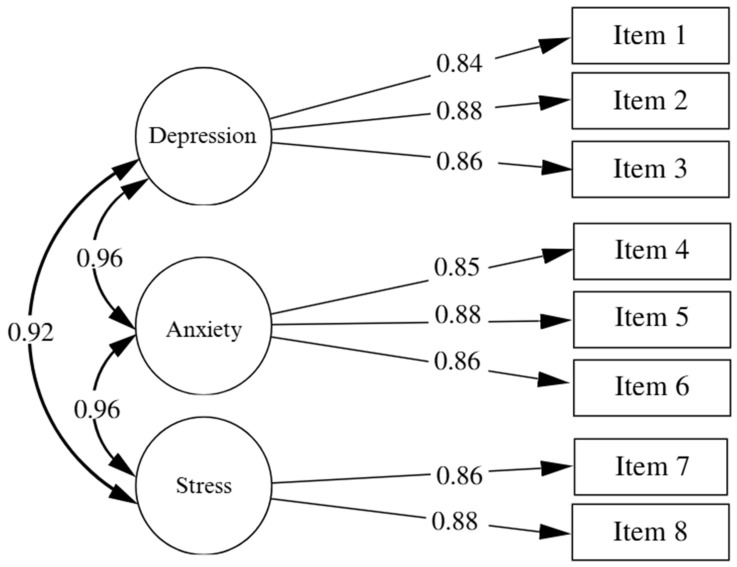
DASS-8 Model.

A confirmatory factor analysis (CFA) was conducted following the methodological criteria recommended in specialized literature (Figure 1). The goodness-of-fit indices obtained demonstrated an excellent fit of the model to the data: χ^2^(17) = 48.000, *p* < 0.001; CFI = 0.99; TLI = 0.98; RMSEA = 0.05 (90% CI: 0.04–0.06); SRMR = 0.01, supporting the adequacy of the proposed factorial structure. Additionally, all factor loadings were statistically significant and greater than 0.50, indicating a strong relationship between the items and their latent factors, thus supporting the structural validity of the instrument. Regarding the internal consistency of the subscales, the results were highly satisfactory. The reliability coefficients calculated included Cronbach’s alpha (α) and McDonald’s omega (ω), with consistent values: for the Depression factor, α = 0.90 and ω = 0.90; for the Anxiety factor, α = 0.90 and ω = 0.90; and for the Stress factor, α = 0.87 and ω = 0.87.

### 3.3. Invarince

Measurement invariance of the scale by gender, stage of adolescence, type of educational institution, and place of residence was assessed using multigroup confirmatory factor analysis, following the hierarchical sequence of models proposed by Meredith (1993) and Cheung and Rensvold (2002). In the configural model, all parameters were freely estimated in each group, requiring only the same factor structure (same pattern of loadings). In the metric model, equality constraints were added to the factor loadings across groups, assuming that the items relate to the factors in the same way. In the scalar model, in addition to the loadings, the intercepts (or thresholds) were constrained to be equal, which allows for the comparison of latent means. Finally, in the strict model, additional equality constraints were imposed on the residual variances, assuming equivalent measurement precision across groups. For gender, the indices supported strict invariance, allowing valid comparisons between girls and boys at all levels (structure, loadings, intercepts, and errors). The CFI of the configural model was 0.984, and the changes in CFI (ΔCFI) for the metric (ΔCFI = 0.002), scalar (ΔCFI = 0.002), and strict (ΔCFI = 0.006) models remained within the accepted threshold (ΔCFI ≤ 0.01), indicating factorial equivalence between groups. With respect to stage of adolescence (early, middle, and late), configural, metric, and scalar invariance were confirmed, allowing for the comparison of latent means across age groups. However, the strict model showed a marked decrease in fit (CFI = 0.931; ΔCFI = 0.022), and strict invariance was therefore not achieved. As a result, comparisons should be limited to the factor structure and latent means, without assuming equality of measurement error across groups. Regarding type of educational institution, strict invariance was supported, with an initial CFI of 0.992 and minimal variation across successive models (ΔCFI ≤ 0.005), which supports full equivalence between students from public and private schools. Finally, for place of residence, configural, metric, and scalar invariance were met, but when the strict model was applied, a substantial decline in fit was observed (CFI = 0.945; ΔCFI = 0.042), exceeding the critical threshold and suggesting that equivalence of measurement errors cannot be assumed between students from urban and rural areas (Table 3).

## 4. Discussion

In the present study, confirmatory factor analysis supported the three-dimensional structure of the 8-item Depression Anxiety Stress Scale (DASS-8) in Peruvian school adolescents, with excellent fit indices. This pattern is consistent with the accumulated evidence supporting the three-factor structure of the DASS-8 in community and clinical adult samples from different countries (Ali et al., 2021, 2022c). Beyond replicating good statistical indices, the central finding is that the model maintains its internal coherence and differentiation between depression, anxiety, and stress in a new context: school-aged adolescents in a Latin American country and within an educational setting. This suggests that the selected items adequately capture the expression of emotional distress at this stage of development, where symptomatology may manifest in specific ways related to academic stress and the psychosocial changes characteristic of adolescence.

The main novelty of this work lies in providing psychometric evidence for the DASS-8 in a Latin American school adolescent population, a group that has been scarcely studied to date, given that most previous research has focused on adults, both in clinical and community samples. For example, the DASS-8 has shown good psychometric properties in psychiatric patients and the general population in Saudi Arabia (Ali et al., 2021), in healthy adults from the United States, Australia, and Ghana (Ali et al., 2022c), in family caregivers of people with dementia in Italy and Switzerland (Ali et al., 2022a), and in women with non-cancer chronic pelvic pain in Australia (Ali et al., 2022b). In this regard, the results of the present study broaden the scope of application of the DASS-8 and show that an ultra-brief version can also be valid and useful in educational contexts, where available time and assessment burden are limited. The excellent model fit may be explained, at least in part, by the clear and concise wording of the items, which facilitates comprehension among adolescents and reduces cognitive load during responding.

With respect to internal reliability, the depression, anxiety, and stress subscales showed excellent and highly balanced levels of internal consistency. In comparative terms, these coefficients fall within the high range and are similar to, or even slightly higher than, those reported in adult studies in community and clinical settings (Ali et al., 2021, 2022a, 2022b, 2022c). This pattern suggests that the DASS-8-S items perform consistently among school adolescents and that the item selection achieves a good balance between brevity and precision. From an applied standpoint, this combination of high reliability and brief format reinforces the usefulness of the DASS-8-S as a screening tool in school settings, where there is a need to rapidly identify students with potentially clinically relevant levels of depression, anxiety, or stress.

The measurement invariance analyses showed that the scale is strictly invariant by gender and by type of educational institution, indicating that the DASS-8-S measures the same constructs with the same structure, loadings, and intercepts in boys and girls, as well as in students from public and private schools. In practice, this allows for latent mean comparisons between these groups with a high degree of confidence, reducing the risk of measurement bias related to gender or school type. In contrast, for stage of adolescence and place of residence, configural, metric, and scalar invariance were achieved, but not strict invariance. This pattern is consistent with previous evidence showing that the more stringent levels of invariance (particularly strict invariance) are sensitive to cultural and demographic differences (Ali et al., 2022a; Chen, 2007). In our case, it suggests that although the structure and meaning of the factors are comparable across developmental stages and between urban and rural contexts, measurement errors may vary, and thus comparisons of observed scores should be interpreted with some caution. Nevertheless, the achievement of scalar invariance in most analyses indicates that it is possible to compare latent means across groups, which is especially relevant for examining mental health inequalities by gender, school type, or residential context in school populations.

When interpreting these results, it is important to take into account the characteristics of the sample and the design. The sample was obtained through non-probability convenience sampling and included school adolescents from a limited set of institutions in Peru. Consequently, inferences should be restricted to contexts with similar characteristics, and claims about generalization to other Latin American countries should be understood as reasonable hypotheses rather than definitive conclusions. Future research should replicate these analyses in probabilistic samples, in different regions of the country and in other countries in the region, as well as in underrepresented rural or Indigenous contexts, in order to strengthen the external validity of the findings and assess the stability of the DASS-8-S structure across diverse sociocultural environments.

Finally, this study makes several contributions to the field. First, it provides robust evidence of the structural validity, reliability, and factorial invariance of the DASS-8-S in Peruvian school adolescents, reinforcing its use as a brief instrument for assessing depressive, anxiety, and stress symptoms in educational settings. Second, it shows that the scale can be used equitably across boys and girls and between public and private institutions, facilitating group comparisons in epidemiological studies and school-based intervention programs. Third, the findings on partial invariance by stage of adolescence and place of residence open avenues for research on how developmental and contextual factors shape the expression and measurement of emotional distress during adolescence. Future work could explore the incremental and predictive validity of the DASS-8-S in relation to other mental health indicators and academic performance, as well as its sensitivity to change in longitudinal studies and psychoeducational interventions aimed at promoting well-being among school students.

### 4.1. Implications

The findings of the present study have important implications for clinical and educational practice, the formulation of public mental health policies, and theoretical development in cross-cultural psychometrics. First, from an applied perspective, the validation of the DASS-8 in Peruvian schoolchildren provides a brief, reliable, and culturally adapted psychometric tool for the early detection of symptoms of depression, anxiety, and stress in school settings. This scale can be used by school psychologists, educational counselors, and community mental health professionals as a rapid and effective screening instrument, allowing for the identification of at-risk students and their referral to timely clinical interventions, even in resource-limited settings. The brevity of the instrument (8 items) facilitates its inclusion in routine assessments within school wellness programs or as part of protocols for the prevention of emotional problems, without generating a significant burden for respondents or technical teams. From the perspective of public policy, the availability of a validated scale for the Peruvian adolescent population strengthens epidemiological surveillance systems for school-age mental health. Educational institutions can incorporate the DASS-8-S into their strategies for monitoring students’ psychological well-being at a national level, enabling the collection of comparable, longitudinal, and georeferenced data. Moreover, since the instrument demonstrated factorial invariance by gender and type of educational institution, psychometric equity in assessment is ensured, allowing fair comparisons of scores between male and female students, as well as between students from public and private schools. This is particularly relevant for targeted policies aimed at vulnerable populations, where it is essential to have diagnostic tools that are sensitive yet fair and unbiased. On a theoretical level, the results contribute to the body of knowledge regarding the dimensional structure and cross-cultural validity of emotional measurement instruments. In particular, it confirms that the tripartite structure of the DASS-8, originally proposed for adults, also fits appropriately in the adolescent context within a Latin American culture. This finding reinforces the tripartite model as a solid theoretical framework for understanding emotional distress in youth. However, the absence of strict invariance by stage of adolescence and place of residence (urban vs. rural) suggests that measurement errors may vary according to developmental or sociocultural factors, raising theoretical questions about the stability of emotional measurement throughout adolescent development and across different cultural environments.

### 4.2. Limitations

Although this study provides solid evidence regarding the validity and reliability of the DASS-8 in Peruvian schoolchildren, it has some limitations. First, the cross-sectional design prevents the establishment of causal relationships or the evaluation of the temporal stability of the instrument. Future longitudinal studies will allow the examination of its sensitivity to change and consistency over time.

Second, the non-probabilistic convenience sampling limits the generalizability of the results. It is recommended that future research employ probabilistic sampling to better represent the Peruvian school population, including rural regions and indigenous communities. Additionally, other relevant variables such as socioeconomic status, ethnic background, or access to mental health services were not considered, even though they could influence the expression of symptoms and item comprehension. Another limitation is the exclusive use of self-reports, which may introduce bias. Future studies should incorporate multiple sources of information (e.g., parents, teachers, or clinical evaluations).

Finally, although the three-dimensional structure was confirmed through CFA, alternative models such as the bifactor model were not explored. Comparative studies could offer new perspectives on the best factorial representation of the DASS-8 in adolescents.

## 5. Conclusions

Although this study provides solid evidence for the validity and reliability of the DASS-8 in Peruvian school students, it has some limitations. First, the cross-sectional design precludes establishing causal relationships or assessing the temporal stability of the instrument. Future longitudinal studies will be necessary to examine its sensitivity to change and consistency over time. Second, the use of non-probability convenience sampling limits the generalizability of the findings. It is recommended that future research employ probability sampling strategies to better represent the Peruvian school population, including rural regions and Indigenous communities. Moreover, other relevant variables such as socioeconomic status, ethnic background, and access to mental health services were not considered, even though they may influence the expression of symptoms and the interpretation of items. Another limitation is the exclusive use of self-report measures, which may introduce bias. Future studies should incorporate multiple sources of information (parents, teachers, or clinical assessments). Finally, although the three-dimensional structure was confirmed through CFA, alternative models such as bifactor models were not explored. Comparative studies could offer new insights into the most appropriate factorial representation of the DASS-8 in adolescents.

## Figures and Tables

**Table 1:** Sociodemographic Characteristics.

Characteristics		n	%
Gender	Girls	396	49.4
	Boys	405	50.6
Adolescence	Middle	227	28.3
	Late	269	33.6
	Early	305	38.1
Type of Institution	Private	243	30.3
	Public	558	69.7
Place of Residence	Rural	170	21.2
	Urban	631	78.8
Academic Level	Primary	81	10.1
	Secondary	720	89.9
Year of Study	Fourth Year	48	6.0
	First Year	200	25.0
	Fifth Year	136	17.0
	Second Year	226	28.2
	Sixth Year	38	4.7
	Third Year	153	19.1

**Table 2:** Descriptive Statistics.

DASS-21 Items	English	Spanish	M	SD	g^1^	g^2^	r(i-tc)
	*Depression*	*Depresión*					
Item 10	1. I felt that I had nothing to look forward to.	Sentí que no tenía nada por lo cual emocionarme.	1.14	1.19	0.53	−1.26	0.79
Item 13	2. I felt down-hearted and blue.	Me sentí desanimado y triste.	1.17	1.15	0.53	−1.18	0.84
Item 16	3. I was unable to become enthusiastic about anything.	No podía entusiasmarme por nada.	1.08	1.2	0.62	−1.19	0.82
	*Anxiety*	*Ansiedad*					
Item 9	4. I was worried about situations in which I might panic and make a fool of myself.	Estaba preocupado por situaciones en las que podría entrar en pánico y hacer el ridículo.	1.1	1.22	0.58	−1.3	0.82
Item 15	5. I felt I was close to panic.	Sentí que estaba cerca del pánico.	1.08	1.19	0.63	−1.16	0.85
Item 20	6. I felt scared without any good reason.	Me sentí asustado sin ninguna razón válida.	0.98	1.18	0.78	−0.98	0.83
	*Stress*	*Estrés*					
Item 8	7. I felt that I was using a lot of nervous energy.	sentí que estaba gastando mucha energía mental y emocional.	1.02	1.2	0.69	−1.13	0.81
Item 12	8. I found it difficult to relax.	Me resultó difícil relajarme.	1.05	1.17	0.67	−1.08	0.83

**Note:** M = Mean, SD = Standard Deviation; g^1^ = Skewness, g^2^ = Kurtosis; r(i-tc) = Corrected Item-Total Correlation; α = Reliability.

**Table 3:** Measurement Invariance.

Invariance	χ^2^	df	*p*	TLI	RMSEA	SRMR	CFI	ΔCFI
Gender								
Configural	79.124	34	<0.001	0.973	0.058	0.021	0.984	
Metric	89.544	39	<0.001	0.974	0.057	0.023	0.982	0.002
Scalar	97.802	44	<0.001	0.975	0.055	0.024	0.980	0.002
Strict	122.173	52	<0.001	0.973	0.058	0.025	0.974	0.006
Adolescence								
Configural	148.913	51	<0.001	0.937	0.085	0.025	0.962	
Metric	174.443	61	<0.001	0.939	0.083	0.031	0.956	0.006
Scalar	191.965	71	<0.001	0.944	0.080	0.033	0.953	0.003
Strict	263.807	87	<0.001	0.933	0.087	0.038	0.931	0.022
Type of Institution								
Configural	55.623	34	0.011	0.986	0.040	0.016	0.992	
Metric	63.091	39	<0.01	0.987	0.039	0.018	0.991	0.001
Scalar	72.864	44	<0.01	0.986	0.040	0.020	0.989	0.002
Strict	93.619	52	<0.001	0.983	0.045	0.022	0.984	0.005
Place of Residence								
Configural	61.412	34	<0.01	0.985	0.045	0.018	0.991	
Metric	76.446	39	<0.001	0.982	0.049	0.027	0.988	0.003
Scalar	82.969	44	<0.001	0.984	0.047	0.027	0.987	0.001
Strict	221.544	52	<0.001	0.940	0.090	0.040	0.945	0.042

## Data Availability

The data supporting the findings of this study are not publicly available due to privacy and ethical restrictions re-lated to research involving minors. De-identified data may be made available upon reasonable request from the corresponding author.
